# Enhanced Internalization of Nanoparticles Following Ionizing Radiation Leads to Mitotic Catastrophe in MG-63 Human Osteosarcoma Cells

**DOI:** 10.3390/ijms21197220

**Published:** 2020-09-30

**Authors:** Roxana Cristina Popescu, Mihai Straticiuc, Cosmin Mustăciosu, Mihaela Temelie, Roxana Trușcă, Bogdan Ștefan Vasile, Adina Boldeiu, Dragoş Mirea, Radu Florin Andrei, Constantin Cenușă, Laurenţiu Mogoantă, George Dan Mogoșanu, Ecaterina Andronescu, Mihai Radu, Marlon R. Veldwijk, Diana Iulia Savu

**Affiliations:** 1 Department of Life and Environmental Physics, “Horia Hulubei” National Insitute of Physics and Nuclear Engineering (IFIN-HH), 30 Reactorului Street, 077125 Magurele, Romania; roxana.popescu@nipne.ro (R.C.P.); cosmin@nipne.ro (C.M.); mihaela.temelie@nipne.ro (M.T.); mradu@nipne.ro (M.R.); 2 Department of Science and Engineering of Oxide Materials and Nanomaterials, “Politehnica” University of Bucharest (UPB), 1-7 Polizu Street, 011061 Bucharest, Romania; ecaterina.andronescu@upb.ro; 3 Department of Applied Nuclear Physics, “Horia Hulubei” National Insitute of Physics and Nuclear Engineering (IFIN-HH), 30 Reactorului Street, 077125 Magurele, Romania; mstrat@nipne.ro (M.S.); dragos.mirea@nipne.ro (D.M.); radu.andrei@nipne.ro (R.F.A.); 4 Department of Science and Engineering of Oxide Materials and Nanomaterials, National Research Center for Micro and Nanomaterials, “Politehnica” University of Bucharest (UPB), 313 Splaiul Independenţei, 060042 Bucharest, Romania; truscaroxana@yahoo.com (R.T.); bogdan.vasile@upb.ro (B.Ș.V.); 5 Laboratory of Nanobiotechnology, National Institute for Research and Development in Microtechnologies (IMT), 12A Erou Iancu Nicolae Street, 077190 Bucharest, Romania; adina.bragaru@imt.ro; 6 Department of Physics, Applied Science Faculty, “Politehnica” University of Bucharest (UPB), 303 Splaiul Independentei, 060042 Bucharest, Romania; 7 Radioisotopes and Radiation Metrology Department, “Horia Hulubei” National Insitute of Physics and Nuclear Engineering (IFIN-HH), 30 Reactorului Street, 077125 Magurele, Romania; constantin.cenusa@nipne.ro; 8 Research Center for Microscopic Morphology and Immunology, University of Medicine and Pharmacy of Craiova (UMFCV), 2 Petru Rareș Street, 200349 Craiova, Romania; editor@rjme.ro; 9 Department of Pharmacognosy & Phytotherapy, Faculty of Pharmacy, University of Medicine and Pharmacy of Craiova (UMFCV), 2 Petru Rareș Street, 200349 Craiova, Romania; mogosanu2006@yahoo.com; 10 Department of Radiation Oncology, Universitätsmedizin Mannheim (UMM), Medical Faculty Mannheim, University of Heidelberg, 68167 Mannheim, Germany

**Keywords:** MG-63 osteosarcoma, ionizing radiation, iron oxide nanoparticles, doxorubicin, combined antitumor treatment

## Abstract

This study aims to investigate whether ionizing radiation combined with doxorubicin-conjugated iron oxide nanoparticles (NP-DOX) improves the internalization and cytotoxic effects of the nano-carrier-mediated drug delivery in MG-63 human osteosarcoma cells. NP-DOX was designed and synthesized using the co-precipitation method. Highly stable and crystalline nanoparticles conjugated with DOX were internalized in MG-63 cells through macropinocytosis and located in the perinuclear area. Higher nanoparticles internalization in MG-63 cells previously exposed to 1 Gy X-rays was correlated with an early accumulation of cells in G_2_/M, starting at 12 h after treatment. After 48 h, the application of the combined treatment led to higher cytotoxic effects compared to the individual treatment, with a reduction in the metabolic capacity and unrepaired DNA breaks, whilst a low percent of arrested cells, contributing to the commitment of mitotic catastrophe. NP-DOX showed hemocompatibility and no systemic cytotoxicity, nor histopathological alteration of the main organs.

## 1. Introduction

Osteosarcoma is a very aggressive common primary bone tumor affecting predominantly children, teenagers and young adults aged 10–30 years, accounting for 5% of all pediatric malignancies [1].

Current standard management of osteosarcoma consists in multi-modal treatment including surgery, multi-agent chemotherapy (including doxorubicin (DOX)) and radiotherapy, yielding to 60% of the 5-year survival rate, while less than 20% of patients with metastatic disease or relapsed osteosarcoma reach a long-term survival [2,3]. However, none of these conventional treatments are effective in combating the tumor progression, and continuing high rates of limb amputation, pulmonary metastasis and normal tissue toxicity remain unresolved issues [4]. Thus, new effective and safe strategies, in particular targeted therapies, are clearly required [4].

In this context, nanoparticle-based drug delivery systems can be designed to pass biological barriers and to target cancerous cells, where they enter and accumulate to reach the drug therapeutic target, while, in the meantime, reducing its systemic toxicity [5,6]. Iron oxide nanoparticles proved to be suitable for such applications due to their biocompatibility for healthy tissues [7] and magnetic targeting ability [8,9]. Moreover, there are several FDA (Food and Drug Administration)-approved systems based on iron oxide nanoparticles for clinical use in cancer treatment through magnetic hyperthermia [10].

The efficient delivery and retaining of nanoparticles in tumor cells are the most important steps towards clinical translation, with many studies referring to targeting a certain state of the tumor cell, such as G_2_/M cell cycle arrest, which would imply a higher internalizing efficiency [11,12,13]. Previous reports proved that both nanoparticles and radiotherapy can induce cell cycle arrest in G_2_/M phase [14,15,16]. Moreover, it was shown that therapeutic doses of irradiation can improve the uptake of nanoparticles in various cell lines [17], however, this range of irradiation can also lead to side effects. By reducing the ionizing radiation dose, the side effects are also reduced, while the G_2_/M delay can still be induced with the chance of enhancing nanoparticles uptake. Starting from these considerations, we hypothesize that medium doses (1 Gy) of ionizing radiation, followed by a novel nano-system, doxorubicin-conjugated iron oxide nanoparticles (NP-DOX) administration, would enhance the nanoparticles internalization and consequently, would increase the cytotoxic effects of nano-carrier-mediated drug delivery in aggressive tumor cell models, such as the MG-63 human osteosarcoma. Doxorubicin was selected as a chemotherapeutic model due to its fluorescence property, which confers a convenient traceability to the nano-system.

To test our hypothesis, mechanistic investigation concerning the effect of ionizing radiation on the internalization and cytotoxicity of NP-DOX have been performed in a MG-63 human osteosarcoma cell model. We synthesized the nanoparticles (as described in Popescu et al. [18]) and then characterized them using relevant physico-chemical methods (Supplementary Materials Sections S1.1 and S2.1). In vitro testing on MG-63 cells revealed information on the internalization, cyto- and geno-toxicity of NP-DOX and have been used to select the experimental conditions, concentrations and observation time points for the investigations we report here (Supplementary Materials Section S2.2).

The effect of combined ionizing radiation with nanoparticles (1 Gy X-rays applied prior to NP-DOX treatment) was investigated through the metabolic and proliferator behavior of osteosarcoma cells (3-(4,5-dimethylthiazol-2-yl)-2,5-diphenyltetrazolium bromide (MTT), trypan blue and colony formation assays), but also through the genotoxic response (comet and micronuclei assays). Quantitative measurements of the atomic Fe concentration in the cells were done using Particle-Induced X-ray Emission (PIXE) spectrometry [19]. These evaluations were correlated with cell cycle measurements and the possible cell death mechanisms were determined through morphological investigations.

For the first time, our results showed that prior ionizing radiation exposure of MG-63 human osteosarcoma cells determined an enhanced internalization of doxorubicin-conjugated nanoparticles, leading to mitotic catastrophe.

## 2. Results

### 2.1. Cells’ Morphology, Cytoskeleton Evaluation and Doxorubicin-Conjugated Iron Oxide Nanoparticles NP-DOX Internalization

**Figure 1:**
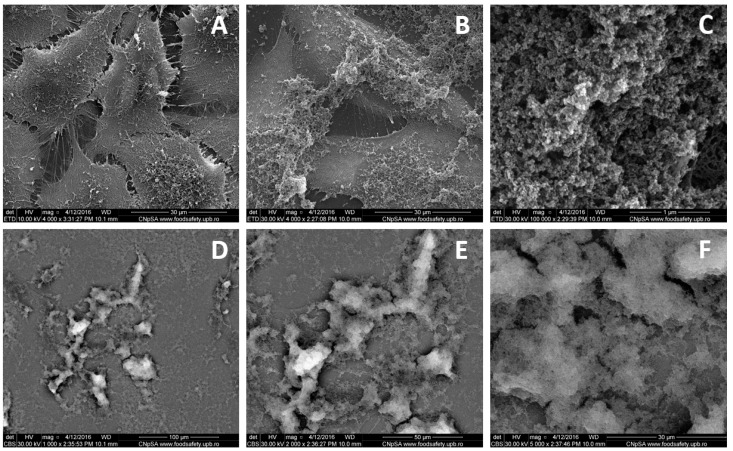
Scanning electron microscopy (SEM) micrographs of MG-63 cells cultured during 24 h: control (**A**) and in the presence of 500 ppm doxorubicin-conjugated iron oxide nanoparticles (NP-DOX) (**B**–**F**). Information was acquired from secondary electrons (**A**–**E**), respectively back scattered electrons (**D**–**F**). Images are acquired at different magnifications: 1000× (**D**), 2000× (**E**), 4000× (**A**,**B**), 5000× (**F**) and 100,000× (**C**).

**Figure 2:**
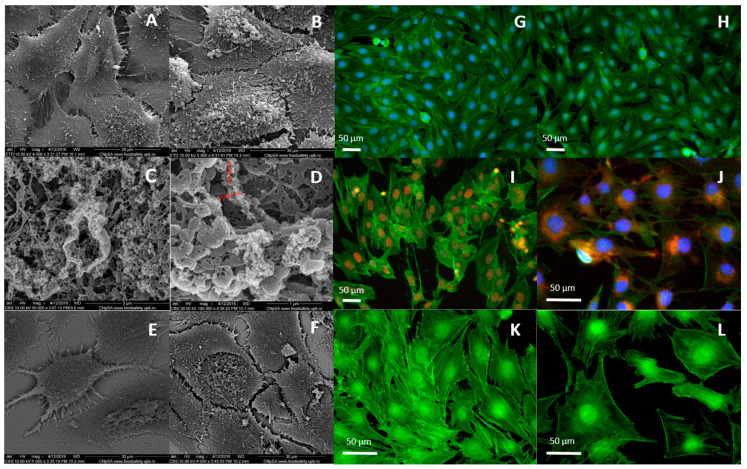
Scanning electron microscopy (SEM) images of MG-63 cells at 48 h of culture: control (**A**,**E**) and in presence of 500 ppm NP-DOX (**B**–**D**,**F**). Information was acquired from secondary electrons (**A**–**D**), respectively back scattered electrons (**E**,**F**). Images were acquired at different magnifications: 4000× (**A**,**F**), 5000× (**B**,**E**), 50,000× (**C**) and 100,000× (**D**). Fluorescence microscopy images of cells at 48 h of culture in presence/absence of nanoparticles: (**G**) control cells (0 ppm nanoparticles), (**H**) cells exposed to 500 ppm NP, (**I**) 2 ppm free DOX (equivalent to DOX concentration in 500 ppm NP-DOX), (**J**) cells exposed to 500 ppm of bare NP-DOX. Immunohistochemical staining of actin filaments in MG-63 cells exposed during 48 h to (**K**) control and (**L**) 500 ppm NP-DOX. Green: Phalloidin-Fluorescein-5-isothiocyanate (FITC), blue: Hoechst, red: Doxorubicin.

Scanning electron microscopy (SEM) was used to evaluate the detailed morphology of NP-DOX-treated MG-63 osteosarcoma cells and their interaction with the nanoparticles (Figure 1 and Figure 2). The images acquired for cells exposed to 500 ppm nanoparticles for 24 h (Figure 1) and 48 h (Figure 2A–F) revealed a severely altered appearance and an increase in total volume, compared to control cells. At 24 h, the whole membrane appeared to be covered with a layer of agglomerated nanoparticles (Figure 1B,C). The entire analyzed surface was coated by a thin organic layer, including nanoparticles, as shown through back scattering acquisition mode (Figure 1D,E). At 48 h after treatment (Figure 2), less nanoparticle aggregates appeared on the surface of the exposed MG-63 cells (Figure 2B), probably due to internalization, as indicated by images in back scattering mode (Figure 2F). The nanoparticles-treated osteosarcoma cells showed a similarly altered morphology (Figure 2B). Aggregates of NP-DOX directly interacted with the cellular membranes and the elongations of the actin filaments in the extracellular medium, inducing a ruffled aspect (Figure 2C,D). Vesicle-like structures were emphasized on the exterior of the MG-63 cells treated with 500 ppm nanoparticles (Figure 2B–D).

Fluorescence imaging was employed to evaluate the internalization and localization of free DOX, NP and NP-DOX in MG-63 osteosarcoma cells, for the highest equivalent concentrations used in the study, and to detect morphological alterations of the cells by the preceding treatment (Figure 2). Bare NP up to concentrations of 500 ppm did not cause any morphological alterations of the MG-63 cells after 48 h of exposure (Figure 2H). Treatment with DOX (Figure 2I) and NP-DOX (Figure 2J) in equivalent concentrations induced a modification of their cellular structure and morphology. The osteosarcoma cell volume increased as a result of cells’ density decrease (Figure 2I,J). In addition, actin filaments lost their defined fibril structure (Figure 2H,J,L). Free DOX was completely located in the nucleus of the cell (Figure 2I), while DOX-conjugated nanoparticles were situated in the cytoplasm of the cells, in the peri-nuclear area (Figure 2J).

### 2.2. Prior Radiation Significantly Enhanced the Cyto- and Geno-Toxic Effect Induced by NP-DOX

The potential cytotoxic effects of prior ionizing radiation and NP-DOX on MG-63 human osteosarcoma cells were evaluated through metabolic (MTT assay), proliferation (MTT assay and Trypan blue cell counting) and genotoxic investigations (micronucleus and comet assays).

Confluent MG-63 cells were irradiated with 1 Gy, grown for 4 h and were subsequently exposed to different concentration of NP-DOX in the range of 0–500 ppm.

**Figure 3:**
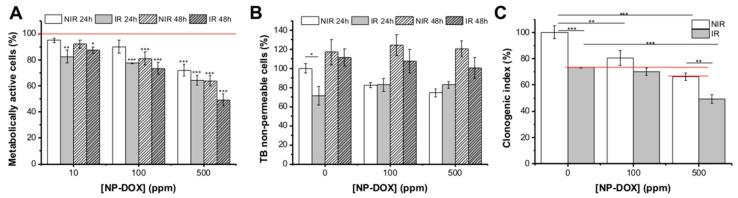
Viability of MG-63 cells exposed to NP-DOX in equivalent concentrations for 24 h and 48 h. One group of cells was previously exposed to 1 Gy X-ray (ionizing radiation (IR)) vs. non-irradiated controls (NIR). Evaluation through: (**A**) metabolic activity measurements, (**B**) membrane permeabilization, (**C**) clonogenic survival. Data are presented as mean ± standard error of the mean (SEM); * 0.01 < *p* < 0.05, ** 0.001 < *p* < 0.01, *** *p* < 0.001.

Cells showed a significant reduction in MTT metabolic ability, accentuated for groups that received both treatments (Figure 3A). Thus, cells that underwent 1 Gy X-Ray and NP-DOX exposure, showed a statistically significant reduction in the tetrazolium salt reduction ability in MG-63 cells, dependent on the nanoparticles concentration (22.64% ± 0.23% (*p* < 0.001) for 100 ppm, 35.67% ± 53.67% (*p* < 0.001) for 500 ppm at 24 h and 26.54% ± 4.4% (*p* < 0.001) for 100 ppm, 50.77% ± 54.45% (*p* < 0.001) for 500 ppm at 48 h, compared to control cells).

Trypan blue assay revealed that the number of viable cells decreased after 24 h of treatment (compared to seeded cell number), as a result of an initial cytotoxic effect of nanoparticles and/or radiation treatment (Figure 3B). However, measurements after 48 h of treatment showed that the cells’ proliferation was not totally suppressed, as the total viable cell number increased, compared to corresponding samples at 24 h.

Clonogenic assay was done to assess the long-term cytotoxicity of prior radiation treatment (0 Gy, 1 Gy) and NP-DOX (0, 100 and 500 ppm) (Figure 3C). The cell survival decreased with radiation treatment (a reduction of 26.73% ± 0.6% as compared to untreated cells), with the effect being accentuated by the addition of 500 ppm nanoparticles for 48 h (total reduction of 50.62% ± 5.8% as compared to untreated control). NP-DOX alone had an inhibiting effect on the MG-63 survival, dependent on the nanoparticles’ concentration. Thus, for 100 ppm, the reduction of survival is of 19.51% ± 9.5%, and for 500 ppm, the reduction is of 33.59% ± 4.75%, compared to control cells. An important significant effect (*p* < 0.001, respectively 0.001 < *p* < 0.01) of ionizing radiation (1 Gy X-rays) and the NP-DOX (500 ppm) treatment on MG-63 clonogenic survival fraction with regard to the single treatment (radiation or nanoparticles) is evident.

**Figure 4:**
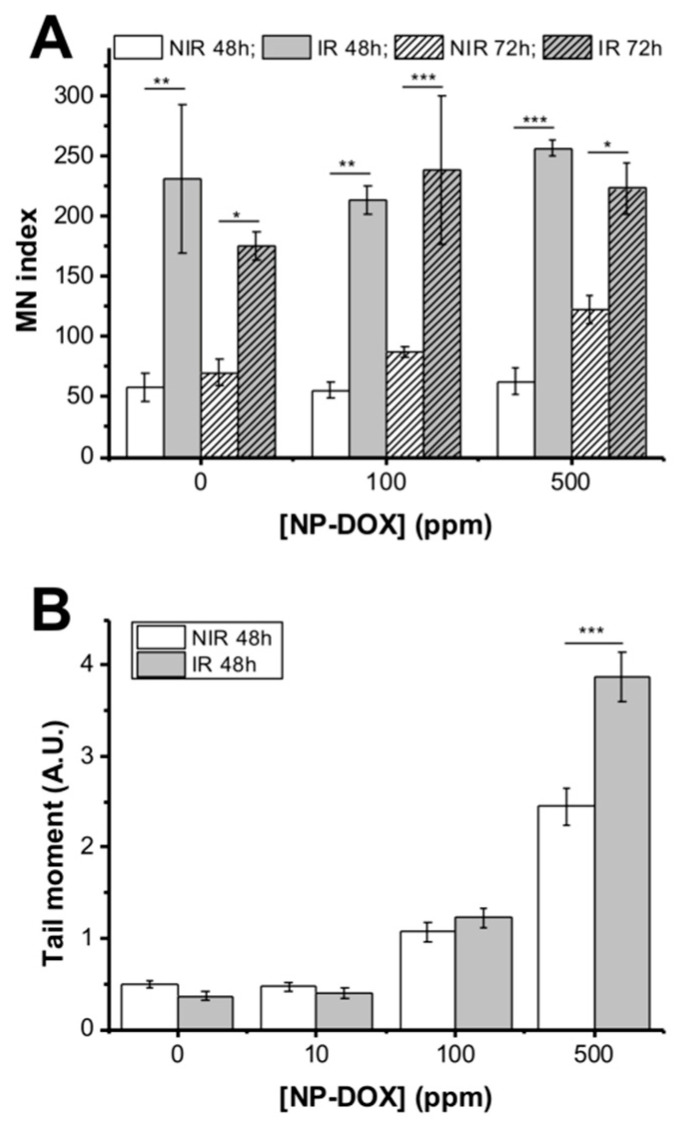
(**A**) Micronuclei in MG-63 cells, non-irradiated or irradiated with 1 Gy and exposed to NP-DOX for 48 and 72 h. (**B**) DNA breaks measured using alkaline comet assay for MG-63 cells either non-irradiated or irradiated with 1 Gy and exposed for 48 h to NP-DOX. Data are presented as mean ± SEM. * 0.01 < *p* < 0.05, ** 0.001 < *p* < 0.01, *** *p* < 0.001.

The micronuclei measurement was done at 48 and 72 h of treatment, respectively (Figure 4A). The NP-DOX exposure alone did not show any statistically significant induction of micronuclei in MG-63 cells at any of the time points and concentrations employed. As expected, irradiation alone induced chromosome fragmentation, demonstrated by a statistically significant increase in micronuclei at 48 h (*p* < 0.01), and at 72 h (*p* < 0.05). In the1 Gy X-ray + nanoparticles groups, the number of micronuclei increased, compared to control (untreated groups). However, NP-DOX did not determine an additional effect to radiation, but rather the prior exposure to the1 Gy X-ray induced a statistically significant effect compared to groups exposed only to nanoparticles (*p* < 0.01 for 100 ppm, *p* < 0.001 for 500 ppm at 48 h, *p* < 0.001 for 100 ppm and *p* < 0.05 for 500 ppm at 72 h).

However, comet assay showed that the DNA breaks increased with NP-DOX concentration and irradiation at 48 h (Figure 4B). The exposure of MG-63 cells to 500 ppm nanoparticles after 1 Gy X-ray determined a 3.01-fold increase in the measured tail intensity (*p* < 0.001), compared to control. In contrast with the micronucleus assay, prior radiation induced a statistically significant effect in DNA breaks compared to NP-DOX alone for 500 ppm groups (*p* < 0.001) with respect to control groups, where radiation alone produced no effect.

### 2.3. Radiation Enhanced NP-DOX Internalizing in MG-63 Cells, Due to Early Induction of G_2_/M

**Figure 5:**
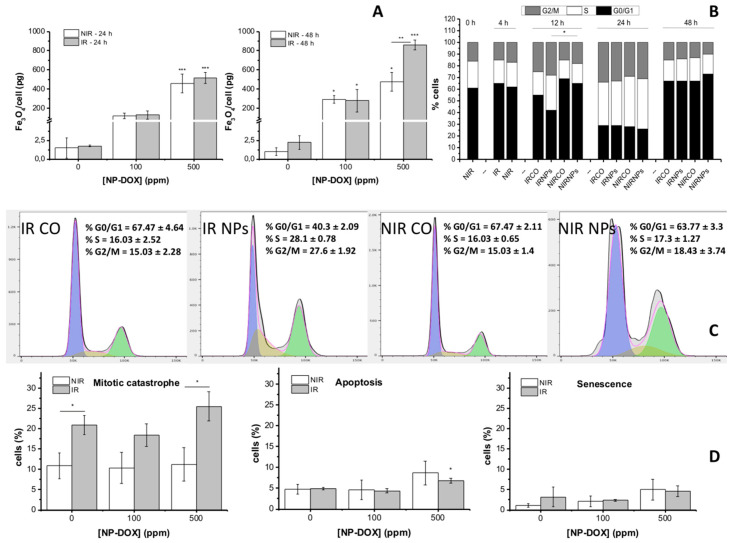
(**A**) Quantity of internalized NP-DOX in MG-63 cells exposed to different concentrations of NP-DOX for 24 and 48 h. One group of cells was previously exposed to 1 Gy X-ray. Cell cycle distributions of MG-63 cells that were treated with and without X-ray irradiation (0 vs. 1 Gy) and nanoparticles (0, 500 ppm NP-DOX) at (**B**) 12 h and (**C**) 0–48 h, respectively. (**D**) Cell death induced by 1 Gy X-rays, 100 ppm and/or 500 ppm, NP-DOX, in MG-63 cells. Cells were treated for 48 h and reseeded during 24 h for morphological evaluation and classification as apoptotic, multinuclear or senescent. Data are presented as mean ± SEM; * 0.01 < *p* < 0.05, ** 0.001 < *p* < 0.01, *** *p* < 0.001.

We investigated the mechanisms induced by ionizing radiation on the internalization of NP-DOX in MG-63 cells using quantitative measurements of the atomic Fe concentration that were correlated with cell cycle measurements (Figure 5A).

The PIXE technique was used to measure the atomic concentration of Fe inside the MG-63 cells cultivated in the presence of NP-DOX for 24 and 48 h, in groups previously exposed to different radiation doses (0 and 1 Gy) (Figure 5A). Results showed that exposure to NP-DOX induced an increase in atomic Fe concentration proportional to the fed nanoparticles concentration. Thus, after 24 h of exposure to 500 ppm NP-DOX, a concentration of 458.61 ± 97.12 pg/cell of Fe_3_O_4_ (*p* < 0.001, compared to control) was measured, while in the equivalent group that received prior radiation treatment, a higher concentration (517.3 ± 57.15 pg/cell, *p* < 0.001, compared to control) was determined. After 48 h, a concentration of 473.5 ± 97.63 pg/cell (*p* < 0.05, compared to control) internalized nanoparticles were measured for non-irradiated MG-63 cells exposed to 500 ppm NP-DOX, and 860.15 ± 52.36 pg/cell (*p* < 0.001, compared to control) nanoparticles for the group exposed to 1 Gy X-ray and 500 ppm NP-DOX, respectively. Thus, after 48 h, the prior exposure to 1 Gy X-ray determined a statistically significant increase in the internalized amounts of nanoparticles in MG-63 cells for the highest given concentration (500 ppm), with *p* < 0.01 compared to the equivalent non-irradiated group.

MG-63 cells were cultured until confluence to enhance the percent of cells in G_0_/G_1_ phase and afterwards received 1 Gy X-ray treatment. Subsequent splitting and reseeding did not cause any statistically significant changes in the cell cycle distribution during 4 h of cultivation (Figure 5B,C). Twelve hours after radiation exposure, obvious changes in the cell cycle of the cells in each group were observed. 1 Gy X-ray treatment caused an accumulation of cells in G_2_/M after 12 h (24.8% ± 2.3%, *p* < 0.05, compared to control cells), while nanoparticles alone did not cause a statistically significant effect. However, the group that received both radiation and NP-DOX treatment showed a significantly higher percent of cells accumulated in G_2_/M phase (27.6 ± 1.92, *p* < 0.01, compared to control cells, and *p* < 0.05 compared to non-irradiated NP-DOX-treated cells). At 24 h, about 30% of the cells underwent G_2_/M in all subjected groups (no statistically significant difference). At 48 h, the percent of cells in G_2_/M was reduced in all groups (no statistically significant difference between the groups), showing that cells probably escaped G_2_/M blockage and entered mitosis.

### 2.4. Cells Exit G_2_/M Arrest and Undergo Mitotic Catastrophe after Exposure to X-ray Irradiation and Subsequent NP-DOX Treatment

The type of cell death after prior radiation and NP-DOX treatment of MG-63 cells was investigated through morphological determinations of the cell death (i.e., apoptosis, mitotic catastrophe, senescence).

The results (Figure 5D) showed that, after 48 h of NP-DOX treatment and another 24 h of resistant clones’ sub-culturing, there is no additional statistically significant induction of apoptosis, for any of the subjected groups, with the exception of the group treated with 1 Gy X-ray and 500 ppm NP-DOX, where 6.8% ± 0.06% of cells showed apoptotic alterations (*p* < 0.05, compared to irradiated control). Irradiation alone determined an increase of 1.92-fold in cells showcasing multi-nuclei (*p* < 0.05, compared to control), while for groups receiving only nanoparticles treatment, no statistically significant alteration in number of multi-nucleated cells was observed. On the contrary, for cells exposed to 1 Gy X-ray and 500 ppm NP-DOX, an increase of 2.34-fold was observed (*p* < 0.01, compared to non-irradiated control). The radiation treatment and nanoparticles seemed to contribute to the induction of a statistically significant increase of the mitotic catastrophe events for the highest NP-DOX concentration (*p* < 0.05). A small percent of senescent cells was observed in the populations exposed either to the highest concentration of nanoparticles or to the combined treatment.

Our results showed that MG-63 cells experience mitotic catastrophe in case of combined treatment (1 Gy of X-rays and NP-DOX). At 48 h, cells undergoing both treatments were released from G_2_/M phase (low percent of cells in G_2_/M, similar to control), with unrepaired DNA (high amount of DNA breaks) and aberrant mitosis (presence of multi-nucleated cells).

## 3. Discussion

In this report, we successfully proved that prior exposure to medium levels of ionizing radiation enhanced the internalization and the cytotoxicity of the novel synthesized nano-construct NP-DOX in aggressive MG-63 human osteosarcoma cells. Firstly, we designed, synthesized and characterized the nano-system (Supplementary Information Sections S1.1 and S2.1). Further, we demonstrated an efficient uptake of NP-DOX through macropinocytosis and transport in the peri-nuclear area. The MG-63 cells’ treatment with the DOX-loaded nanoparticles led to a significant decrease in cells’ proliferation, while the genotoxicity increased (Supplementary Information, Section S2.2). Next, we tested and showed that the combined ionizing radiation-nanoparticles (1 Gy irradiation applied prior to NP-DOX) treatment is more efficient in the induction of a cytotoxic anti-proliferative effect in osteosarcoma cells compared to nanoparticles alone. Mechanistic insights concerning the effect of ionizing radiation on the internalization and cytotoxicity of NP-DOX in the MG-63 osteosarcoma cells were revealed. A significantly higher percent of DOX-conjugated nanoparticles in the cells previously exposed to 1 Gy X-ray compared to other groups was found. This was accompanied by a premature entry of the MG-63 cells in G_2_/M phase. However, cells re-entered in G_1_ at 48 h after treatment (similar to untreated cells) and underwent mitotic catastrophe.

The bare iron oxide nanoparticles have been synthesized (Supplementary Information, Section S1.1) using a modified chemical co-precipitation method, as in Popescu et al. [18], which implied a 1.65:1 Fe^3+^:Fe^2+^ ratio, in order to compensate the oxidation of Fe^2+^ [20,21]. The biocompatibility of bare Fe_3_O_4_ nanoparticles (NP) was proven by our in vitro data on cyto- and geno-toxicity (Supplementary Information, Section S2.2 and Figure 2H), confirming other reports [7].

The conjugation of doxorubicin with the Fe_3_O_4_ nanoparticles (NP-DOX) was done using an in situ functionalizing method, as the organic phase was introduced into the reaction system directly into the precipitation medium and was present during the nucleation of the nanoparticles. This allowed a direct interaction between the chemotherapeutic substance and the bare NP. It has been previously reported that doxorubicin can form complexes with Fe, by means of the hydroquinone moieties, resulting in strong linking of the chemotherapeutic to iron [22]. Our data confirms that a stable interaction of the organic and inorganic phases was established. Nevertheless, the presence of the organic phase was confirmed through thermogravimetric analysis, as a difference of weight loss during heating of NP-DOX compared to non-conjugated NP (Supplementary Information, Section S2.1), but also through the prolonged fluorescence of the nano-constructs, as evidenced due to doxorubicin intrinsic fluorescence (Figure 2J).

Our investigations through fluorescence microscopy showed that conjugated nanoparticles are distributed in the cytoplasm of the MG-63 cells, most probably in endosome structures, while free doxorubicin directly went into the nucleus of the cells, due to a passive diffusion transport [23]. The mechanism of internalizing for NP-DOX is different and takes place more slowly. Thus, after 48 h of incubation, doxorubicin-conjugated nanoparticles are located in the vicinity of the nucleus. Kamba et al. have observed that CaCO_3_/doxorubicin nanoparticles were predominantly spread in the cytoplasm of the MG-63 osteosarcoma cells, compared to free doxorubicin, which directly diffused into the nucleus of the cells [24], with the effect being attributed to the trapping of the constructs into the endosome compartment of the cells.

A possible mechanism of internalizing is macropinocytosis, as suggested by SEM investigations, emphasized through the “ruffling” aspect of the cellular membrane, but also the presence of macropinosomes (Figure 1 and Figure 2C,D) [25,26]. Moreover, the extracellular fibrils network matches the description of the *Candida glabrata* internalizing in MG-63 cells and imaging of Gd nanoparticles macropinocytosis in head and neck squamous cell carcinoma cells (SQ20B) [27]. As macropinocytosis is directly related to actin cytoskeleton dynamics, information on actin filaments’ organization was correlated with high-resolution SEM morphology observations. At 24 h after treatment, the fibrils actin network underwent morphological alterations matching the description of micropinocytosis-specific “ruffling” (Figure 1 and Figure 2C,D) [26,27,28]. However, at 48 h after treatment, there was less structure in the actin network and the cytoplasm seemed to have lost its integrity (Figure 2). This might be due to DOX release leading to actin filaments’ depolymerisation (Figure 2I,J,L), as bare NP did not cause any alteration of the actin filaments’ structure (Figure 2H) [29,30].

Further we have used medium-dose X-ray (1 Gy) to sensitize confluent MG-63 osteosarcoma cells for the newly developed doxorubicin-conjugated iron oxide nanoparticles. We showed that combined treatment enhanced the cytotoxic and genotoxic effects in osteosarcoma cells compared to nanoparticles alone (Figure 3 and Figure 4). Our data also showed that G_2_/M temporary cell cycle arrest in MG-63 cells exposed to combined treatment (1 Gy X-ray + 500 ppm nanoparticles) was linked to an enhanced internalization of NP-DOX (Figure 5). The group of cells receiving 1 Gy and 500 ppm nanoparticles showed an early induction of G_2_/M arrest at 12 h that reached a maximum at 24 h after treatment, when all groups underwent G_2_/M arrest (Figure 5B,C). A comparable prolonged effect of G_2_/M cell cycle arrest was reported for HeLa Hep2 cells exposed to 2.5 Gy (60 MeV, 0.45 Gy/min) [31]. More recently, other similar results have been reported by Yi et al., who have shown that CuS@Melanin/polyethylene glycol PEG-DOX-loaded nanoparticles might have good potential in the nanoparticles-mediated chemo-radiotherapy, proving both a radio-sensitizing effect in post-internalization radiotherapy or enhanced uptake in previously irradiated cells [17]. Liu et al. [13] have also observed that a previous exposure of HeLa cells to ionizing radiation can lead to an enhanced quantity of gold nanoparticles to be internalized, an effect linked to a higher percent of cells undergoing cell cycle arrest in G_2_/M following X-ray exposure.

It is well known that ionizing radiation causes a cell cycle arrest, determining cells to have a non-reproductive state [32]. On the other hand, the internalized agent itself can cause an effect, leading to a G_2_/M arrest and, thus, to its enhanced uptake by the cell, with some examples of such events being the formation of reactive oxygen species [33] or cell cycle interfering drug release (e.g., doxorubicin [34], docetaxel [35]). In our case, during the first 24 h of exposure, more than 60% of DOX is released from the nanoparticles. Although it is known for DOX to cause G_2_/M arrest in MG-63 cells [36], the released amount during this period of time from the internalized nanoparticles might be too small to cause a significant alteration of G_2_/M on its own.

Previously, it has been stated by many groups that cells’ uptake is strongly influenced by their cell cycle phase [37,38,39,40]. A significant difference in internalizing among cells in different phases of the cell cycle can be noticed after 24 h (mostly one complete cell cycle for most types of immortalized cells) [33,38]. Taking this into consideration, cells that go through more or extended G_2_/M phases during a monitored time interval will exhibit a higher concentration of internalized nanoparticles [13].

Exposure to ionizing radiation (1 Gy) prior to NP-DOX administration led to a significantly increased concentration of nanoparticles per cell in the 1 Gy + 500 ppm group, measured at 48 h. Before this time point, the previously irradiated cells have been undergoing a prolonged G_2_/M phase, allowing more nanoparticles to be internalized. A higher ability of nanoparticles’ uptake in an osteosarcoma tumor model was also proven in vivo for liposomal doxorubicin (Caelyx, 8 mg/kg dose) [41]. Caelyx was intravenously administered one day before irradiation (single dose 8 Gy or fractionated doses of 3.6 Gy for 3 days, 6 MV X-rays), leading to an improved penetration of DOX inside the osteosarcoma in irradiated subjects.

At 48 h (when all cells exit G_2_/M), a significant increase in chromosomal damage was observed for the 1 Gy + 500 ppm NP-DOX group, compared to the 500 ppm group, while there were small differences in inhibition of proliferation between the two groups. Long-term monitoring (clonogenic assay) showed that previous radiation induced a statistically significant reduction of the survival rate for cells receiving NP-DOX treatment. However, the combined treatment effect is additive rather than synergic since the sum of the effects of each factor is a bit higher than the combined effect (Figure 3C).

Many cancer cells, including MG-63, exhibit the inability of the cells to undergo apoptosis, resulting in continuous proliferation, even if their DNA was damaged [42,43]. Syljuasen et al. [44] have proved that in human osteosarcoma cells, a G_2_ checkpoint arrest occurs, induced by exposure to ionizing radiation, correlated with DNA breaks (measured by gamma H2AX assay); however, the cells are able to exit this temporary arrest and undergo mitosis at around 30h from radiation treatment, while still expressing the markers for unrepaired DNA double-strand breaks. Our results show that all cells exit from G_2_/M arrest until 48 h, being in agreement with Syljuasen’s report. In cells irradiated in G_0_/G_1_ and exiting G_2_/M arrest to enter mitosis, DNA double-strand breaks have duplicated and can be counted as micronuclei in M phase cells [45].

The ability of cells exhibiting DNA damage to enter mitosis without fulfilling DNA repair in G_2_ has been defined as mitotic catastrophe and described repeatedly [46,47]. The aberrant mitosis leads to either death or polyploidy, the cells eventually undergoing cell death, senescence or formation of single tetraploid cells [46,48]. After 48 h of treatment, classification of MG-63 cells by morphologic clonogenic assay showed that neither irradiation alone, nor nanoparticles alone, resulted in any statistically significant apoptotic death after induction of mitosis (Figure 5A). However, exposure to 500 ppm NP-DOX after 1 Gy X-rays showed a statistically significant increase in the population of apoptotic cells, compared to only irradiated cells. Polyploidy was shown to be determined by radiation treatment and enhanced by the addition of the highest concentration of NP-DOX. Additionally, the previously mentioned observations on actin network reorganization in MG-63 cells after 48 h of treatment matches older descriptions on actin filaments’ reorganization and implication in mitotic catastrophe cell death [49,50,51]. It is for the first time that the mitotic catastrophe was correlated with an increased accumulation of nanoparticles after exposure to ionizing radiation.

Furthermore, the hemocompatibility evaluation and in vivo biodistribution studies emphasized that the NP-DOX did not cause any systemic response, nor organ cytotoxicity (Supplementary Material, Section 2.3 and Section 2.4), which encourage further exploitation of these nano-systems in tumor preclinical models.

## 4. Materials and Methods

### 4.1. Materials

NP and NP-DOX nanoparticles were obtained using a modified chemical co-precipitation method, as in Popescu et al. [18]. A detailed description of nanoparticles’ synthesis process and characterization is presented in Supplementary Information Sections S1.1 and S2.1.

### 4.2. Cell Culture

MG-63 human osteosarcoma cell line purchased from ATCC^®^ was used as in vitro tumor model for the nano-constructs’ cytotoxicity assessment. The cells were cultured in MEM Earle’s (MEM) (Biochrom, Merck Milipore, Darmstadt, Germany), supplemented with 10% fetal bovine serum (Biochrom, Merck Milipore), 1% L-glutamine (Biochrom, Merck Milipore), 1% non-essential amino-acids (Sigma Aldrich Germany) and 1% antibiotics (penicillin and streptomycin) (Biochrom, Merck Milipore). Cell cultures were maintained at 37 °C in a humidified incubator (95% air, 5% CO_2_).

### 4.3. Cells Morphology, Cytoskeleton Evaluation and NP-DOX Internalization

Scanning electron microscopy: SEM was performed in order to obtain information about the interaction of the nanoparticles with the cellular membrane. For this purpose, 50,000 cells were seeded on 24 mm diameter glass slides, placed in 6-well plates and cultured for 24 h under standard conditions, to allow attachment. The cells were treated with 1000 µL of nanoparticle suspensions in complete culture medium at the concentration of 500 ppm, while in control wells (untreated cells), complete culture medium was added. The samples were incubated for another 14 and 48 h, respectively. Then, the slides were washed with phosphate buffered saline(PBS), fixed in 2.5% glutaraldehyde for 1 h and were dehydrated in ethanol (EtOH) and hexamethyldisilasane (HMDS) solutions (EtOH in MiliQ 70%, 90% and 100% for 30 min; HMDS in EtOH 25%, 50% and 100% for 3 min). The samples were covered with a thin layer of Au and analyzed using a FEI SEM (Hillsboro, OR, USA) with a beam of secondary electrons having energies up to 30 keV.

Immunofluorescence: For the qualitative evaluation of the cytotoxic effects and nanoparticle internalization, we employed a multi-fluorescence labeling, enabling the cells’ nucleus, cytoskeleton and the nanoparticles’ localization. For the nanoparticles’ localization, the fluorescent properties of doxorubicin have been exploited. The cell seeding and nanoparticles treatment was done similar as for SEM. To prepare the slides for fluorescence microscopy, the medium with nanoparticles was removed from the wells and the cells were gently washed with PBS 3 times. Then, 3.7% paraformaldehyde solution was used for fixing, during 10 min. Next, the cells were permeabilized with 1% tryton X for 10 min and stained with fluorescein-5-isothiocyanate(FITC)-Phalloidin for 40 min and Hoechst for 10 min (in dark). The cells were gently washed with PBS after each step. The visualization of the prepared samples was done using an Olympus IX71 fluorescence microscope (Tokyo, Japan) and the image recording was done using an iXON^EM^+ DU-897 camera (Andor Technology, Belfast, UK). Appropriate filter sets were used to record separate images for nuclei (blue), actin filaments (green), DOX and NP-DOX (red), in the same area. The images were merged using ImageJ (National Institutes of Health, Bethesda, MD, USA).

### 4.4. Irradiation

250,000 MG-63 cells were seeded into T25 flasks and incubated in standard conditions of temperature and humidity until they reached confluence. The cell culture medium was replaced with fresh medium and the cells were exposed to a dose of 1.085 Gy X-rays, at a debit of 2.394 mGy/s, during 8 min. For the generation of X-rays, an XStrahl XRC 160 (Surrey, UK) source was used (energy of 150 keV, current of 20 mA). 30 min after radiation, cells were detached and reseeded as described below. The cells were allowed to attach for 4 h; afterwards, the medium was replaced with fresh medium containing nanoparticles (concentrations of 0, 10, 100, 500 ppm) and incubated in standard conditions of temperature and humidity during different time intervals (24, 48 or 72 h, according to the assays described below).

### 4.5. Cytotoxicity and Proliferation Measurements

The quantitative evaluation of the cytotoxic effect of the nanoparticles was done using the tetrazolium-salt (MTT)-based viability assay, according to Mosmann et al. and Denizot et al. [52,53]. After undergoing radiation treatment, the cells were re-seeded at a concentration of 4000 cells/well in 96-well plates and allowed attachment for 4 h. The nanoparticle treatment was added at this time and the viability measurements were done at 24, 48 and 72 h after the treatment. The absorbance at 570 nm was determined using a Mithras LB 940 (Berthold Technologies) microplate reader.

Trypan blue staining was used to evaluate the percent of dead cells or proliferation after subsequent nanoparticles exposure. The cells that underwent radiation were re-seeded at a concentration of 500,000/well and incubated during 4 h. Afterwards, the nanoparticles were added (concentrations of 100 ppm and 500 ppm NP-DOX) and incubated for different time intervals (12, 24, 48 h). At the specified timepoints, the cells were washed with PBS and detached using 0.1% trypsin, which was then inactivated using 1:3 complete MEM. A volume of the cells’ suspension was mixed with equal volume of trypan blue and cells were counted using a hemocytometer.

Clonogenic assay: The cells that underwent irradiation treatment were re-seeded and treated as for Trypan blue staining. After 48 h incubation with nanoparticle treatment, cells were harvested and counted. 300 cells/well from each group were seeded into 6-well plates in 2 mL and cultured for 14 days. After this time, the samples were gently washed with PBS and fixed with 3.7% paraformaldehyde (PFA) during 10 min. Then, the cells were stained using a 0.5% Giemsa solution and colonies comprising at least 50 cells were counted.

Genotoxicity assessment: The chromosomal damage was assessed using the micronucleus assay [54,55]. After undergoing radiation treatment, 30,000 cells/25 mm slide were seeded and allowed attachment for several hours. Solutions of NP-DOX in different concentrations were added (0, 100 and 500 ppm) and incubated for an additional 48 or 72 h. Cytochalasin B was added, and cells were incubated for an additional 18h. The slides were fixed using a 1:9 acetic acid:methanol solution and the DNA was stained using acridine orange (10 µg/mL final concentration). The counting was done as previously reported [55].

The comet assay was used to measure the DNA breaks after radiation and/or NP-DOX treatment. Irradiated MG-63 cells were re-seeded and treated as for the clonogenic assay. The samples were harvested after 48 h of NP exposure. The comet assay was done in alkaline conditions, similarly to the procedure described by Singh et al. [56]. The cells were suspended in complete culture medium, mixed with 1% low melting point agarose (Sigma Aldrich) and the suspension was layered on the surface of the slides previously covered with 1% normal melting agarose (Sigma Aldrich). The slides were immediately covered with cover slips and incubated for 5 min on ice to allow the solidification of the agarose. The cover slips were removed, and cold lysis was performed using a 2.5 M NaCl, 100 mM ethylenediaminetetraacetic acid EDTA, 10 mM Tris (pH = 10), dimethyl sulfoxide DMSO and 1% tryton X-100 for 1 h at 4 °C. Then, the DNA electrophoresis was performed for 25 min, using an alkaline electrophoresis solution (300 mM NaOH, 1 mM EDTA, pH = 13, at 4 °C). Then, the DNA was neutralized using a 0.4 M Tris solution, at pH = 7.5. The DNA was stained using 70 µL ethidium bromide 20 µg/mL, washed with deionized water and covered with cover slips. Analysis of comet images was performed using the software Comet Assay IV, Perceptive Instruments, UK, and the fluorescence microscope Olympus (BX51), Olympus Optical Co., Tokyo, Japan, equipped with a Marlin F-046 CC digital camera.

Morphological evaluation of cell death (Mitotic catastrophe, apoptosis and senescence evaluation): The samples were seeded and treated as for the clonogenic assay. After 48 h of interaction with NP-DOX, 10,000 cells/25 mm glass slide were seeded and cultured for an additional 24 h. The cells were fixed using 1:9 solution of acetic acid:methanol and stained using acridin orange. In order to evaluate the clonogenic cell death, the cells were counted and grouped into normal, apoptotic, undergoing mitotic catastrophe or senescence according to Kobayashi et al. [57].

### 4.6. Quantitative Cellular Internalizing Using Particle-Induced X-ray Emission

Cells that underwent radiation treatment were detached and reseeded (500,000 cells/well). The nanoparticle treatment was done as for the clonogenic assay. After 24 and 48 h respectively, of incubation in the presence of nanoparticles, the cells were gently washed with PBS 3 times, for 5 min, in order to remove all the non-internalized NP. Afterwards, they were detached using 1% trypsin and fixed using 2.5% glutaraldehyde. The samples were carefully deposited on a 1 µm thick Mylar foil that was glued on an aluminum frame and allowed to dry.

PIXE analysis was performed at the 3 MV TandetronTM accelerator from “Horia Hulubei” National Institute for Physics and Nuclear Engineering (IFIN-HH), using a 2.7 MeV proton beam and the in-air irradiation setup [58]. The characteristic X-rays were recorded by a silicon drift detector positioned at 45° with respect to the beam direction and the spectra were processed with the Gupixwin 2.2.4 software [59].

The concentration of nanoparticles was calculated by reporting the Fe_3_O_4_ values to the viable cell number obtained from trypan blue assay, at each corresponding timepoint.

### 4.7. Cell Cycle Analysis

The cells that underwent irradiation treatment were seeded and treated as for the clonogenic assay. 30 min prior to fixing, 10 μM bromodeoxyuridine (BrdU) was added into each well. The samples were washed, detached and fixed at 0, 4, 12, 24 and 48 h after irradiation, as in Reference [60]. BrdU-labeled cells were fixed in 70% ice-cold ethanol at −20 °C for at least 24 h. The cells were treated with 2 mol/L HCl/0.5% tryton X-100 for 30 min, then washed in PBS/0.5% bovine serum albumin (BSA) and incubated in 0.1 mol/L sodium tetraborate for 2 min. After an additional wash in PBS/0.5% BSA, cells were incubated with monoclonal anti-BrdU antibody (1:300; BD Biosciences) for 30 min. The cells were washed in PBS/0.5% BSA and then incubated with a FITC-conjugated secondary antibody for 30 min (1:300; Merck). Finally, the cells were stained with 5 µg/mL propidium iodide and 50 µg/mL ribonuclease (RNase) in PBS for 30 min. The assay was performed on the BD FACSLyric (BD Biosciences, Franklin Lakes, NJ, USA), and analyzed using FlowJo 10.5 software (BD).

### 4.8. Statistics

The values were presented as means ± standard error of the mean (SEM). Data were analyzed statistically using one-way analysis of variance (ANOVA).

## 5. Conclusions

Novel doxorubicin-conjugated iron oxide nanoparticles NP-DOX were prepared to improve the internalization and cytotoxicity in aggressive osteosarcoma cells previously exposed to medium-dose ionizing radiation. Highly crystalline nanoparticles were successfully synthesized and efficiently internalized in MG-63 human osteosarcoma cells through macropinocytosis and transport in the peri-nuclear area, showing cytotoxic effects. Previous exposure to ionizing radiation determined an enhanced cytotoxic, anti-proliferative and genotoxic effect of NP-DOX. A significantly higher percent of DOX-conjugated nanoparticles in the cells previously exposed to radiation was accompanied by an early entry of the MG-63 cells in G_2_/M phase. Re-entering in G_1_ at 48 h after treatment (similar to untreated cells) was correlated with morphological observations considering the nuclear alteration of osteosarcoma cells undergoing mitotic catastrophe. The G_2_/M transition appears to be a specific target in the strategy of combining radiotherapy with NP-DOX to increase therapeutic efficacy while sparing side effects. This combination could be envisaged as a potential candidate for osteosarcoma treatment.

## Supplementary Materials

Supplementary materials can be found at https://www.mdpi.com/1422-0067/21/19/7220/s1. Figure S1 SEM for (A) Fe_3_O_4_ and (B) NP-DOX nanoparticles; Figure S2 TEM (A,E), HR-TEM (B,F) and SAED (C,G) for NP (A–C), respectively for NP-DOX (E–G); (D) release kinetics of doxorubicin from NP-DOX; data are presented as mean ± SEM; (H) XRD pattern for (non-) conjugated magnetite; the pattern has peaks characteristic for magnetite (JCPDS number 19-0629) at 18.31° (111), 30.33° (220), 35.51° (311), 43.16° (440), 53.61° (422), 57.17° (511); red-diffraction pattern for NP and blue- diffraction pattern for NP-DOX; Figure S3 Proliferation of MG-63 osteosarcoma cells exposed to NP and NP-DOX in equivalent concentrations for 24, 48 and 72 h; the data were presented as mean ± SEM; * *p* < 0.05; Figure S4 Morphological apoptosis assay measurements for MG-63 osteosarcoma cells exposed to different concentrations of NP-DOX for 24 h; Figure S5 DNA breaks induction (alkaline comet assay) in MG-63 osteosarcoma cells exposed for 48 h to NP or NP-DOX; the data were presented as mean ± SEM; * *p* < 0.05; Figure S6 Transversal section through samples collected at 7 days from mice injected with NP-DOX: (a) brain; (b) liver; (c) myocardium; (d) pancreas; (e) lungs; (f) kidneys; hematoxilin-eosin staining (400× magnification; measure bar of 50 μm); Figure S7 Transversal section through spleen collected at 7 days from mice injected with NP-DOX; hematoxilin-eosin staining, magnification: (a) 100× (measure bar of 100 μm); (b) 200× (measure bar of 50 μm); (c) 400× (measure bar of 25 μm); (d) 1000× (measure bar of 25 μm); Figure S8 Transversal section through samples collected at 14 days from mice injected with NP-DOX: (a) brain; (b) liver; (c) myocardium; (d) pancreas (200×; measure bar 100 μm); (e) lungs; (f) kidneys; hematoxilin-eosin staining (400× magnification; measure bar of 50 μm); Figure S9 Transversal section through spleen collected at 14 days from mice injected with NP-DOX; hematoxilin-eosin staining, magnification: (a) 200× (measure bar of 50 μm); (b) 400× (measure bar of 25 μm); (c,d) 1000x (measure bar of 25 μm); Table S1 DLS and zeta potential measurements for NP-DOX.
