# Supplementary material for: Enhanced Internalization of Nanoparticles Following Ionizing Radiation Leads to Mitotic Catastrophe in MG-63 Human Osteosarcoma Cells
Source: Int J Mol Sci. 2020 Sep 30;21(19):7220. doi: 10.3390/ijms21197220 (PMC7583846; doi:10.3390/ijms21197220)
Supplement: Supplementary file 1 [file ijms-21-07220-s001.pdf]

Supplementary Information

Enhanced Internalization of Nanoparticles following Ionizing Radiation Leads to Mitotic Catastrophe in MG-63 Human Osteosarcoma Cells

Roxana Cristina Popescu[1,2], Mihai Straticiuc[3], Cosmin Mustăciosu[1,2], Mihaela Temelie[1], Roxana Trușcă[4], Bogdan Ștefan Vasile[4], Adina Boldeiu[5], Dragoş Mirea[3], Radu Florin Andrei[3,6], Constantin Cenușă[7], Laurenţiu Mogoantă[8], George Dan Mogoșanu[9], Ecaterina Andronescu[2], Mihai Radu[1], Marlon R. Veldwijk[10], Diana Iulia Savu[1]**

[1] "Horia Hulubei" National Insitute of Physics and Nuclear Engineering (IFIN-HH), Department of Life and Environmental Physics, 30 Reactorului Street, 077125 Magurele, Romania; roxana.popescu@nipne.ro (R.C.P.); cosmin@nipne.ro (C.M.); mihaela.temelie@nipne.ro (M.T.); mradu@nipne.ro (M.R.)

[2] "Politehnica" University of Bucharest (UPB),Department of Science and Engineering of Oxide Materials and Nanomaterials, 1-7 Polizu Street, 011061 Bucharest, Romania; ecaterina.andronescu@upb.ro (E.A.);

[3] "Horia Hulubei" National Insitute of Physics and Nuclear Engineering (IFIN-HH), Department of Applied Nuclear Physics, 30 Reactorului Street, 077125 Magurele, Romania; mstrat@nipne.ro (M.S.); dragos.mirea@nipne.ro (D.M.)

[4] "Politehnica" University of Bucharest (UPB), National Research Center for Micro and Nanomaterials, Department of Science and Engineering of Oxide Materials and Nanomaterials,313, Splaiul Independenţei, 060042 Bucharest, Romania; truscaroxana@yahoo.com (R.T.); bogdan.vasile@upb.ro (B.S.V.)

[5] National Institute for Research and Development in Microtechnologies (IMT), Laboratory of Nanobiotechnology, 12A Erou Iancu Nicolae Street, 077190 Bucharest, Romania; adina.bragaru@imt.ro (A.B.)

[6] "Politehnica" University of Bucharest (UPB),Applied Science Faculty, Department of Physics, 303 Splaiul Independentei, 060042 Bucharest, Romania; radu.andrei@nipne.ro (R.F.A.)

[7] "Horia Hulubei" National Insitute of Physics and Nuclear Engineering (IFIN-HH), Radioisotopes and Radiation Metrology Department, 30 Reactorului Street, 077125 Magurele, Romania; constantin.cenusa@nipne.ro (C.C.)

[8] University of Medicine and Pharmacy of Craiova (UMFCV), Research Center for Microscopic Morphology and Immunology, 2 Petru Rareș Street, 200349 Craiova, Romania; editor@rjme.ro (L.M.)

[9] University of Medicine and Pharmacy of Craiova (UMFCV),Faculty of Pharmacy, Department of Pharmacognosy&Phytotherapy, 2 Petru Rareș Street, 200349 Craiova, Romania; mogosanu2006@yahoo.com (G.D.M.)

[10] University of Heidelberg, Medical Faculty Mannheim, Universitätsmedizin Mannheim (UMM), Department of Radiation Oncology, 68167, Mannheim, Germany

* Correspondence: Marlon.Veldwijk@medma.uni-heidelberg.de (M.R.V.); dsavu@nipne.ro (D.I.S.); Tel.: +49-621-383-3750 (M.R.V.); +40-214-046-134 (D.I.S.)

1. Materials and methods

1.1. Nanoparticles synthesis

For the synthesis of bare and doxorubicin-conjugated iron oxide nanoparticles (NP and NP-DOX), the following substances were purchased from Sigma Aldrich Chemie GmbH (Munich, Germany): ferrous sulfate 7- hydrate ($FeSO_4 \cdot 7H_2O$), ferric chloride ($FeCl_3$), 25% ammonia solution (NH_3) and doxorubicin hydrochloride (DOX). All chemicals were of analytical purity and used with no further purification.

NP and NP-DOX were obtained using a chemical co-precipitation method similar as in [1]. For this, 2 g $FeSO_4 \cdot 7H_2O$ and 1.2 g $FeCl_3$ were dissolved in 400 mL ultrapure water to obtain the precursor solution and 5 mL of 25% $NH_3 \cdot OH$ solution were mixed with 200 mL ultrapure water to obtain the precipitation medium. In order to obtain doxorubicin-conjugated nanoparticles, 100 mg doxorubicin was also dissolved into the ammonia solution. The precursor solution was added drop wise into the precipitation medium, under magnetic stirring. The resulted colloids were magnetically separated and washed several times with ultrapure water.

The sterilization of the resulted NP and NP-DOX nanoparticle suspensions was done using γ irradiation using a ^{60}Co source (SVST Co-60/B irradiator), at a dose higher than 25 kGy.

1.2. Nanoparticles characterization

Scanning electron microscopy analysis (SEM)

SEM was assessed using a FEI electronic microscope, with a beam of secondary electrons having energies up to 30 keV. The samples were placed onto a carbon strip attached to a metallic stand for visualization.

Transmission electron microscopy analysis (TEM)

The transmission electron microscopy was done using a Tecnai ™ G2 F30 S-TWIN HR-TEM (FEI Company, Hillsboro, OR, USA) equipment, having SAED (selected area electron diffraction). The preparation step of the samples consisted in successive dilutions of the nano-colloids in water, by sonication during 15 minutes. The suspension was placed onto a

holey carbon-copper grid and dried for analysis. The equipment was set to transmission mode at 300 kV, with 2Å point resolution and 1Å line resolution.

X-Ray Diffraction Analysis (XRD)

XRD was employed in order to evaluate the samples crystallinity, using a Shimadzu XRD 6000 diffractometer. The scanning was done at room temperature, using a Cu K α =1.056 Å (15 mA and 30kV) and a Bragg angle of 2 θ = 10- 80.

Thermogravimetric analysis (TGA)

TGA was done using a Shimadzu DTG-TA-50H equipment. The samples were placed into platinum crucibles and screened to 200 mesh prior to analysis: after that, the samples were heated with 10 $K\cdot min^{-1}$, from room temperature to 800°C, under flow of 20 mL/min, in dried synthetic air (80% N_2 and 20% O_2).

Dynamic Light Scattering (DLS) and Zeta Potential Measurements

The stability of NP-DOX was evaluated through hydrodynamic diameter and surface charge (zeta potential) measurements, done using a Delsa™ Nano C instrument (Beckman Coulter, Brea, CA, USA) and recorded using the DelsaNano 3.73 software (Beckman Coulter, Brea, CA, USA). The assessment was done for nanoparticle suspensions in ultrapure water (stock solution of 1 mg/mL concentration) and freshly prepared dilutions in complete culture media (MEM Earle's supplemented with 10% fetal bovine serum, 1% L-glutamine, 1% non-essential aminoacids and 1% penicillin-streptomycin) at concentrations of 10, 100, 500 ppm.

Release kinetics

In order to evaluate the release dynamics of the chemotherapeutic substance from NP-DOX, a suspension of nanoparticles was made in different biologically relevant media at the concentration of stock solution (1 mg/mL): Phosphate Buffer Saline (PBS) with pH=7.4, acid PBS pH=6.5 and endosome-like buffer pH = 4.8 (12 mM NaH_2PO_4, 145 mM NaCl, 4.7 mMKCl). The suspensions were incubated for different time intervals in standard conditions of

temperature and humidity (37±2°C, 5±1% CO_2, more than 90% humidity). Supernatant was collected from each sample, by magnetic separation, at each time-point. Afterwards, as a measure of doxorubicin released in the collected probes, fluorescence intensities (490 nm excitation and 593 nm emission wavelengths) were obtained using a Sunrise microplate reader (Life Sciences- Tecan, Männedorf, Switzerland).

1.3.*In vitro* cytotoxicity evaluation of NP-DOX in absence of radiation

Cell culture

MG-63 human osteosarcoma cell line purchased from ATCC® was used as *in vitro* tumor model for the nano-constructs cytotoxicity assessment. The cells were cultured in MEM Earle`s (MEM) (Biochrom, Merck Milipore), supplemented with 10% fetal bovine serum (Biochrom, Merck Milipore), 1% L-glutamine (Biochrom, Merck Milipore), 1% non-essential amino-acids (Sigma Aldrich) and 1% antibiotics (penicillin and streptomycin) (Biochrom, Merck Milipore). Cell cultures were maintained at 37ºC in a humidified incubator (95% air, 5% CO_2).

MTT viability assay

The quantitative evaluation of the cytotoxic effect of the nanoparticles was done using the tetrazolium- salt (MTT)-based viability assay. For this, 4000 cells/ well were seeded in 96 well plates and allowed attachment for 24h. Meanwhile, solutions of NP, and NP-DOX in equivalent concentrations were prepared by ultrasound dispersion/dilution in complete culture medium (concentrations of 10, 100 and 500 ppm NP-DOX/NP) and added to each well. The viability measurements were done at 24, 48 and 72 h after the treatment. The absorbance at 570 nm was determined using a Mithras LB 940 (Berthold Technologies) microplate reader.

Morphological apoptosis assay

Cell death was scored as previously performed by Temelie et al. [2] in order to obtain information on the cell death mechanism that the MG-63 osteosarcoma cells underwent after nanoparticle treatment. For this, 30000 cells/well were seeded into a 24 wells plate and cultured for 24h in standard conditions of temperature and humidity. Thereafter, the culture medium was

removed and replaced with 600 μL of fresh medium containing nanoparticles in different concentrations (0, 10, 100, respectively 500 ppm). The cells were harvested 24h after the treatment with nanoparticles, stained with acridine orange and ethidium homodimer and scored under fluorescence microscope according to their morphology (viable - round green nuclei; early apoptotic - green half moon-shaped nuclei; late apoptotic orange cytoplasm and green nuclei; and necrotic - red cells with round enlarged nuclei).

Comet assay

For the genotoxicity assessment of NP and NP-DOX, 30000 cells/well were seeded into a 24 wells plate and allowed attachment for 4 hours. Afterwards, the culture medium was removed and replaced with 600 μL of fresh medium containing nanoparticles in different concentrations (0, 10, 100 and 500 ppm). The samples were harvested at 24 and 48 h after the treatment with nanoparticles.

The comet assay was done in alkaline conditions, similarly to the procedure described by Singh et al.[3]. The cells were harvested, suspended in complete culture medium, mixed with 1% low melting point agarose (Sigma Aldrich) and the suspension was layered on the surface of the slides previously covered with 1% normal melting agarose (Sigma Aldrich). The slides were immediately covered with cover slips and incubated for 5 minutes on ice to allow solidifying of the agarose. After this, the cover slips were removed and cold lysis was performed using a 2.5 M NaCl, 100 mM EDTA, 10 mM Tris (pH=10), DMSO and 1% tryton X- 100 for1h at 4°C. Then, the DNA electrophoresis was performed for 25 minutes, using an alkaline electrophoresis solution (300 mMNaOH, 1mM EDTA, pH=13, at 4°C). Then, the DNA was neutralized using a 0.4 M Tris solution, at pH=7.5. The DNA was stained using 70 μL ethidium bromide 20 μg/mL, washed with deionized water and covered with cover slips. Analysis of comet images was performed using the software Comet Assay IV; Perceptive Instruments, UK and the fluorescence microscope Olympus (BX51), Olympus Optical Co., Tokyo, Japan equipped with Marlin F-046 CC digital camera.

1.4.*In vivo* biodistribution

The animal studies were approved by the institutional Ethics Committee of the University of Medicine and Pharmacy of Craiova (Approval No. 73/29.05.2014) and was conducted according to national and EU regulations in the field. Male Balb/C mice, 8–10 week-old, weighing 18–22g were used. The *in vivo* biodistribution of the NP and NP-DOX nanoparticles was tested in 12 albino mice, housed in the Animal Care Unit of University of Medicine and Pharmacy of Craiova and were maintained throughout experiments at 22±20 °C, 55±10% humidity and a 12 h light/dark cycle. Mice were fed with standard rodent diets, and received food and water *ad libitum*. Each subject was intraperitoneally injected with 1 mg/mL suspension of nanoparticles in PBS, respectively with physiological serum, for controls. The subjects were divided in two categories, half of them being sacrificed at 7 days after the treatment and the other half at 14 days after the treatment. Animals were anesthetized with ether solution and the main organs were isolatedfor histological examination (brain, liver, heart, pancreas, lungs, kidneys, spleen). The organs were washed in PBS, preserved in formaldehyde for several days, embedded in paraffin blocks and sectioned using a microtome. The samples underwent hematoxilin-eosin staining (Sigma Aldrich) and were analyzed using Nikon Eclipse 55*i* (Nikon Instruments) optical microscope.

1.5.Hemocompatibility evaluation

The hemocompatibility study was approved by the National Institute of Physics and Nuclear Engineering's Ethics Committee (Approval No. 57/10.05.2019) and was carried out in accordance with relevant guidelins and regulation (Declaration of Helsinki).The informed consent was obtained from all the subjects involved in the study. The hemocompatibility of NP-DOX nanoparticles was assessed according to ASTM standard E2524-08 (Standard test method for analysis of hemolytic properties of nanoparticles). Whole blood from three human healthy donors was used. Nanoparticle samples were prepared as described before in Popescu et al., for concentrations of 500, 100, 20, respectively 4 ppm NPs in PBS [1].

2. Results and discussion

2.1.Nanoparticles synthesis and characterization

Bare and doxorubicin-conjugated iron oxide nanoparticles were synthesized using modified chemical co-precipitation routes and their physical and chemical properties were evaluated comparatively, in order to find whether the presence of the organic chemotherapeutic in the nucleation phase of the nanoparticles influenced the final properties of the nano-system.

Nanoparticles morphology was characterized using both SEM and TEM imaging. The SEM micrographs (Figure S1) showed a homogenous aspect of the nanoparticle powders, the iron oxide nanoparticles being organized as aggregates. The physical dimension of each individual core was measured to be 7.48±0.44 nm, these observations being confirmed by TEM analysis (Figure S2A-B, E-F).

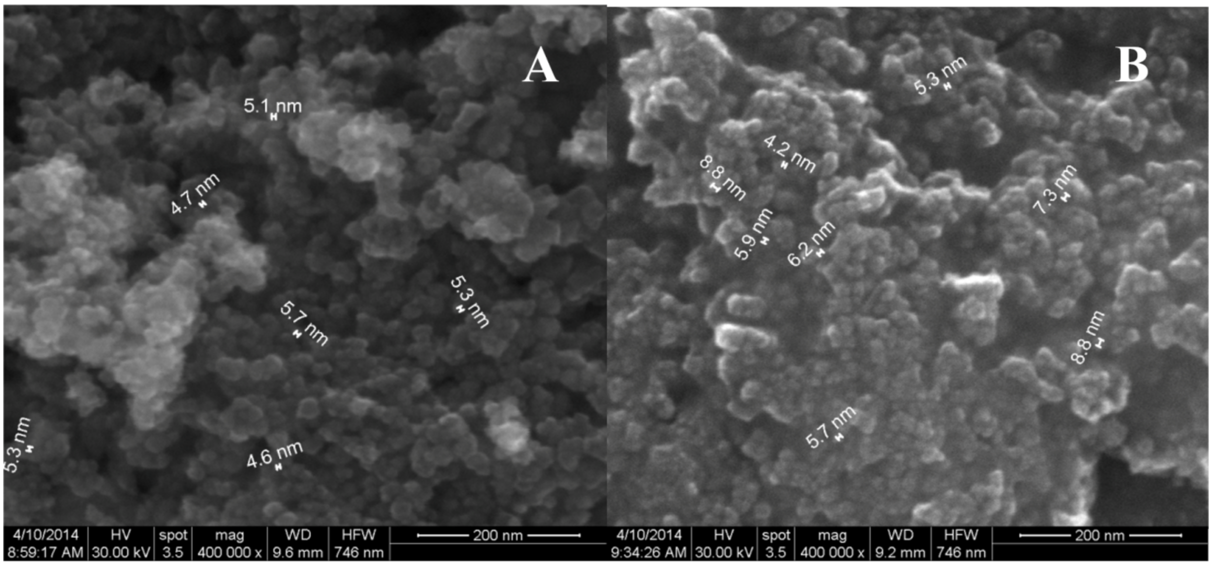

Figure S1. SEM for (A) Fe_3O_4 and (B) NP-DOX nanoparticles.

Both TEM and high resolution TEM (HR-TEM) (Figure S2E-F) helped emphasize the presence of the organic doxorubicin phase in DOX-conjugated nanoparticles, forming a continuous low crystalline mass around the highly crystalline aggregates (Figure S2F, pointed with white arrows), resulting core-shell like structures. The distances between atomic planes in figure S2-B were measured and identified as (220) and (111) for bare and conjugated

nanoparticles [1]. The high degree of crystallinity was confirmed by selected area electron diffraction patterns (SAED)(Figure S2C, G). The SAED spectrums were in concordance with the XRD spectrums, as one can identify the diffraction planes of: (220), (222), (400).[1]

For both constructs, *X-Ray diffraction analysis* (Figure S2H) showed a diffraction pattern characteristic for the mineralogical phase magnetite no other secondary phases being detected [1]. Also, the introduction of the organic doxorubicin into the reaction system did not affect the composition, nor the crystallinity of the samples. Six characteristic diffraction interferences have been identified, the standard 2 θ values with the corresponding diffraction planes: 18.31° (111), 30.33° (220), 35.51° (311), 43.16° (440), 53.61° (422), 57.17° (511), as specified in the standard of JCPDS number 19-0629 [1].

The *quantity of doxorubicin* chemotherapeutic substance interacting with the iron oxide nanoparticles was calculated using the thermogravimetric analysis measurements, through the difference in weight loss after heating bare NP and NP-DOX up to 500°C. Thus, 0.415 wt% of doxorubicin was found in the conjugated sample.

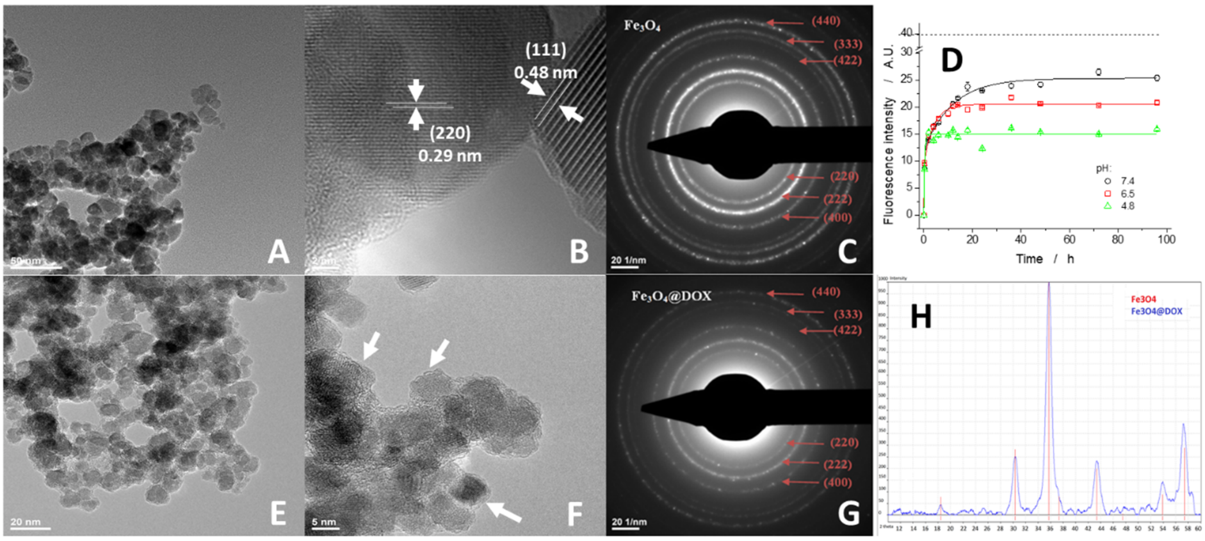

Figure S2. TEM (A, E), HR-TEM (B, F) and SAED (C, G) for NP (A-C), respectively for NP-DOX (E-G); (D) release kinetics of doxorubicin from NP-DOX; data is presented as mean±SEM; (H) XRD pattern for (non-) conjugated magnetite; the pattern has peaks characteristic for magnetite (JCPDS

number 19-0629) at 18.31° (111), 30.33° (220), 35.51° (311), 43.16° (440), 53.61° (422), 57.17° (511); red - diffraction pattern for NP and blue - diffraction pattern for NP-DOX.

The behavior of NP-DOX into the biologic media was studied by means of assessing the *zeta potential and hydrodynamic diameter* of the constructs in complete MEM under standard conditions of temperature and humidity. In this regards, the study was done at concentrations relevant for the *in vitro* biological assays (10, 100, 500 ppm). Highly stable (zeta potential: -36.79 mV) doxorubicin-conjugated iron-oxide nanoparticles had a hydrodynamic diameter of 108.3 nm. Measurements for NP-DOX dilutions in culture media showed a good stability of the negatively charged nano-constructs (-26.67 mV zeta potential for the highest concentration evaluated). Results showed an increase in hydrodynamic size with concentration; the influence of concentration on the Polydispersity Index was not clear, as results showed no proportionality (Table S1).

Table S1. DLS and zeta potential measurements for NP-DOX.

**Sample**	**DLS (nm)**	**PdI**	**Zeta potential (mV)**
Stock solution of NP-DOX	108.3	0.177	-36.79
500 ppm of NP-DOX in complete MEM	278.3	0.200	-26.67
100 ppm of NP-DOX in complete MEM	253.3	0.237	-28.55
10 ppm of NP-DOX in complete MEM	206.2	0.118	-21.99

Release kinetics of doxorubicin in the biological media revealed that the chemotherapeutic substance is gradually enabled from the nano-carrier, under different conditions of pH (7.4, 6.5, 4), temperature and humidity, until 96h of incubation (Figure S2D).

Our data showed that the chemotherapeutic substance was slowly released from the nano-carrier, under physiologic conditions of pH, temperature and humidity, up until 96h of

monitoring (Figure S2D) however, the level of release decreased with pH. This can be explained by the fact that lower pH values of the release medium favor the interaction between the iron oxide nanoparticle and the doxorubicin molecules, preventing their release in the medium. At low pH, DOX has a positive charge due to protonation of the amino group (NH^{3+}) and strongly interacts with the negative O^{-} groups on the surface of iron oxide nanoparticles. This explanation is also supported by the negative values of the zeta potential of Fe_3O_4 nanoparticles (Table S1).

2.2.*In vitro* cytotoxicity evaluation of NP-DOX in absence of radiation

The effect of bare and doxorubicin-conjugated iron-oxide nanoparticles (NP and NP-DOX) on the proliferation of MG-63 human osteosarcoma cells was assessed using the metabolizing efficiency of MTT tetrazolium salt-based assay (Figure S3). At lower time points (24h), the MTT assay was a measure of the metabolizing efficiency of the tetrazolium salts by the tumor cells after their treatment with iron oxide nanoparticles, while, at higher time points (48, respectively 72h) it indicated the proliferation ability of the cells after NPs exposure. Bare NP showed no short-term cytotoxicity in MG-63 cells, for none of the analyzed concentrations, as the viability of exposed cells was close to 100%, compared to untreated cells. However, a slight proliferating activity was observed for the cells exposed to the lowest concentration of NP (10 ppm), the effect increasing with the exposure time (11.17±1.72% increase in viability at 72h, $P<0.001$). The toxicity of MG-63 cells exposed to NP-DOX decreased with concentration and exposure time. For the highest equivalent concentration employed in the study, a fraction of 20.61±1.11% ($P=0.004$) of the cells died in the first 24 h of exposure. The lower concentrations evaluated did not induce any statistically significant reduction of the metabolic activity. After 48h of treatment, 24.73±2.79% ($P=0.003$) of the cells were affected for a concentration of 500 ppm NP-DOX, the effect remaining constant after 72h (a reduction of cell viability with 24.95±0.056%, $P=0.001$, compared to untreated cells). However, lower

concentrations showed an increase in cytotoxicity with10.69±2.54% for 100 ppm, respectively with 10.73±0.29% for 10 ppm at 72h, compared to 48h time point. NP-DOX induced a significant decrease in MG-63 cells viability compared to control group, but also to bare NP in equivalent concentration, as calculated by Student‘s test.

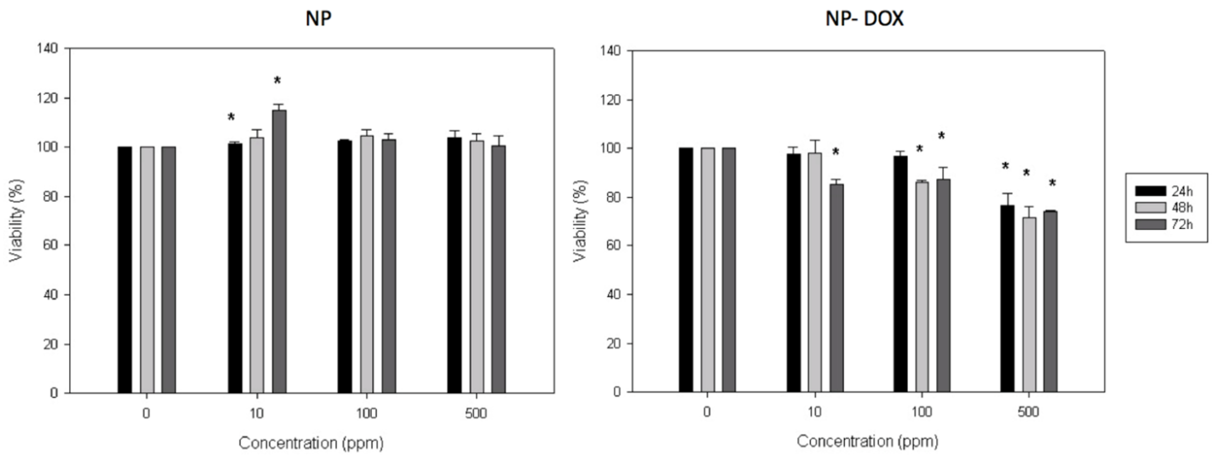

Figure S3. Proliferation of MG-63 osteosarcoma cells exposed to NP and NP-DOX in equivalent concentrations for 24, 48 and72h; the data was presented as mean±SEM; *P<0.05.

The morphological cell death showed a gradual increase in MG-63 apoptotic cells, dependent on concentration of NP-DOX, until 500 ppm, for which 64.2±1.33% (P=0.07) of cells are apoptotic (Figure S4). The number of necrotic cells in the exposed groups did not show any modification compared to the control group.

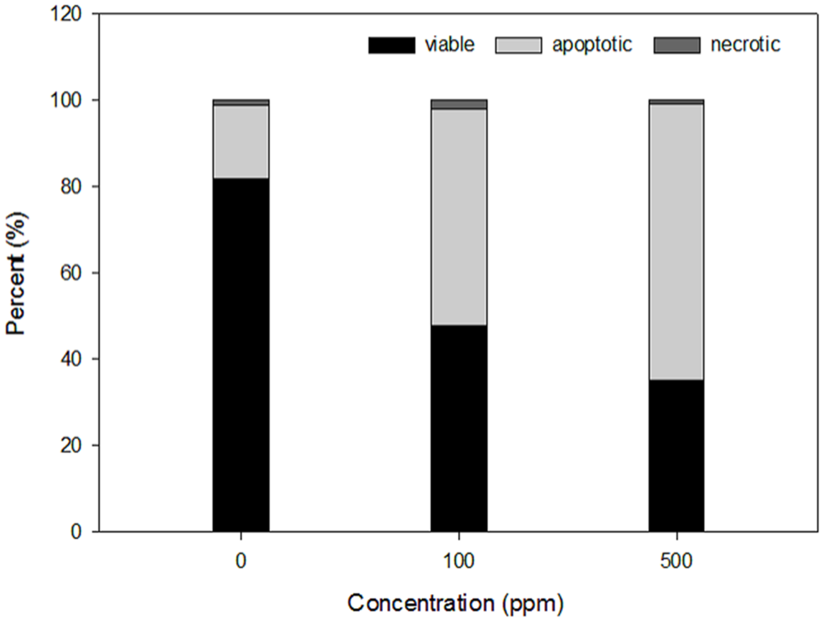

Figure S4. Morphological apoptosis assay measurements for MG-63 osteosarcoma cells exposed to different concentrations of NP-DOX for 24h.

Analysis of the induction of DNA damage (Figure S5) showed that bare iron oxide nanoparticles produced no significantly breaks into the DNA of the cells exposed to NPs for 48h. However, by conjugating doxorubicin to NP, the tail intensity increased by 3.83 folds for the 500 ppm concentration, compared to control samples and was 2.51 folds higher for 100 ppm ($P<0.001$) (Figure S5).

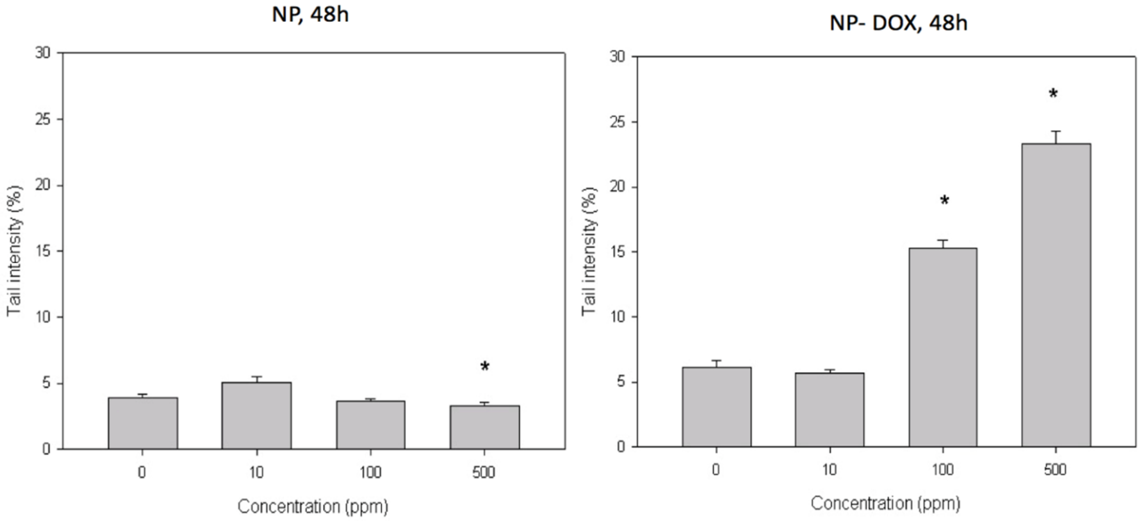

Figure S5. DNA breaks induction (alkaline comet assay) in MG-63 osteosarcoma cells exposed for 48h to NP or NP-DOX; the data were presented as mean±SEM; $*P<0.05$.

2.3.*In vivo* biodistribution of NP-DOX

The biodistribution test in Balb/c mice was done to assess the *in vivo* systemic toxicity of the NP-DOX. The main organs (brain, liver, heart, pancreas, lungs, kidneys and spleen) were harvested at 7, respectively 14 days after treatment and prepared for histological examination using hematoxilin-eosin staining.

Nanoparticles could not be detected at 7 days of administrations at cerebral level, nor in the liver, pancreas, lungs or at renal level (Figure S6). No evident histological alterations of these organs were observed (Figure S6). However, after 7 days of treatment, nanoparticles could be shown in the spleen of the subjected mice, located in the red pulp, in the macrophage cells from the Billroth cordons and sinusoid capillaries (Figure S7). These were displayed as dark brown agglomerated granular structures with ovoid morphology and varied diameters (4.32±0.22 μm) (Figure S7). No nanoparticles could be evidenced in the white pulp of the spleen, but a hypertrophy of it was observed (Figure S7).

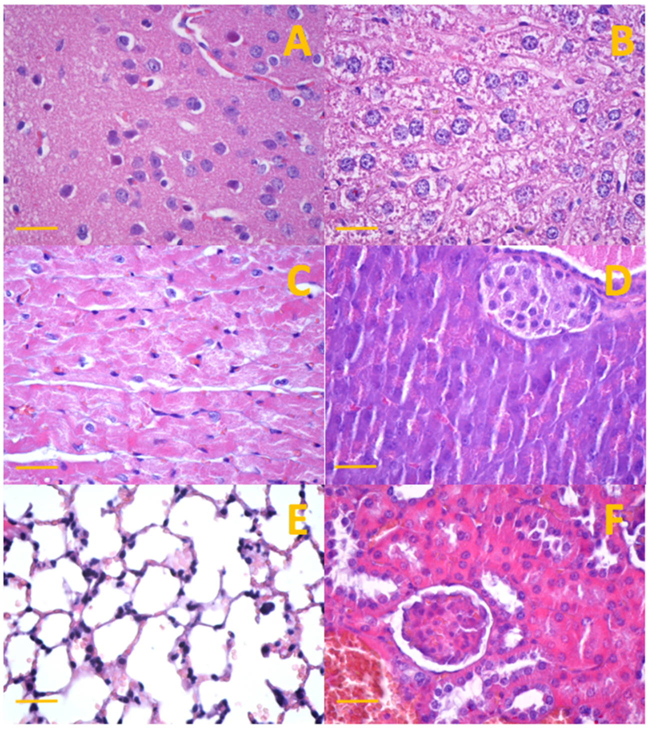

Figure S6.Transversal section through samples collected at 7 days from mice injected with NP-DOX: (a) brain; (b) liver; (c) myocardium; (d) pancreas; (e) lungs; (f) kidneys; hematoxilin-eosin staining (400× magnification; measure bar of 50 μm).

Similar observations were made in the samples harvested at 14 days from administration: no presence of nanoparticles nor histological alterations were highlighted in the main organs (Figure S8), with the exception of spleen (Figure S9), where the NP-DOX were located in the red pulp; also, signs of inflammations were evidenced. Dimensions of NPs aggregates were 2.12±0.07 μm.

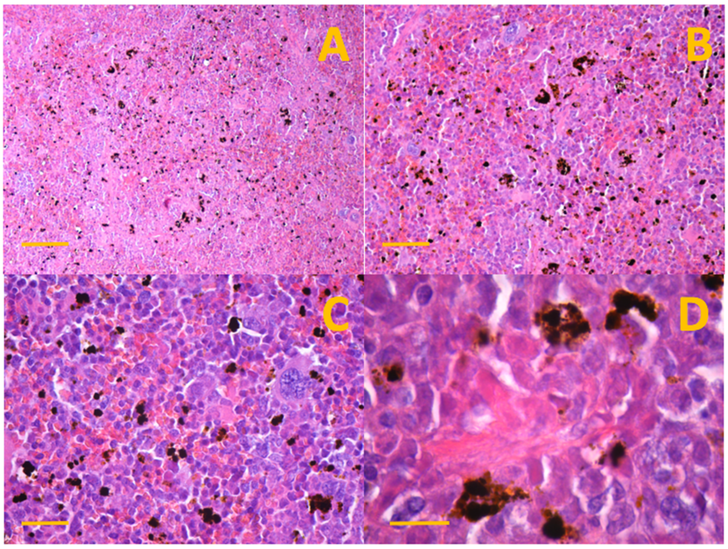

Figure S7.Transversal section through spleen collected at 7 days from mice injected with NP-DOX; hematoxilin-eosin staining, magnification: (a) 100× (measure bar of 100 μm); (b) 200× (measure bar of 50 μm); (c) 400× (measure bar of 25 μm); (d) 1000x (measure bar of 25 μm);

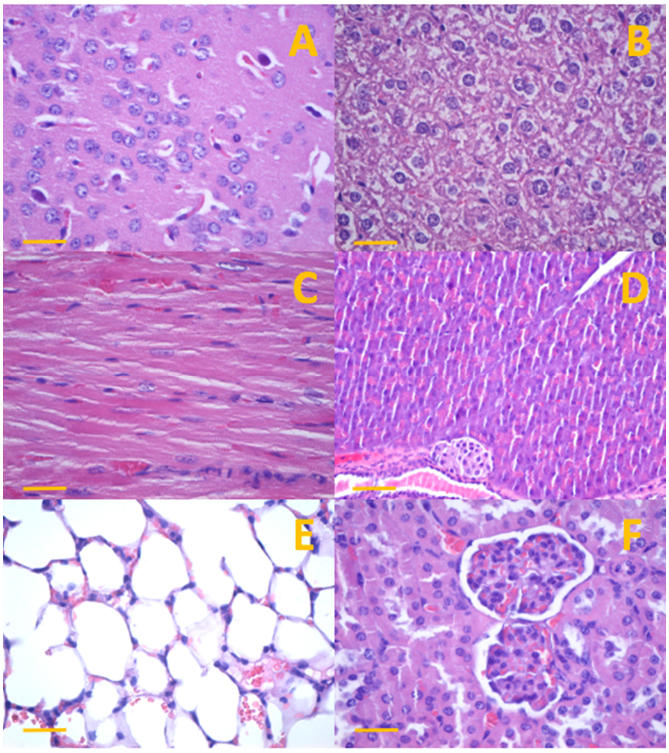

Figure S8.Transversal section through samples collected at 14 days from mice injected with NP-DOX: (a) brain; (b) liver; (c) myocardium; (d) pancreas (200x; measure bar 100µm); (e) lungs; (f) kidneys; hematoxilin-eosin staining (400× magnification; measure bar of 50 µm).

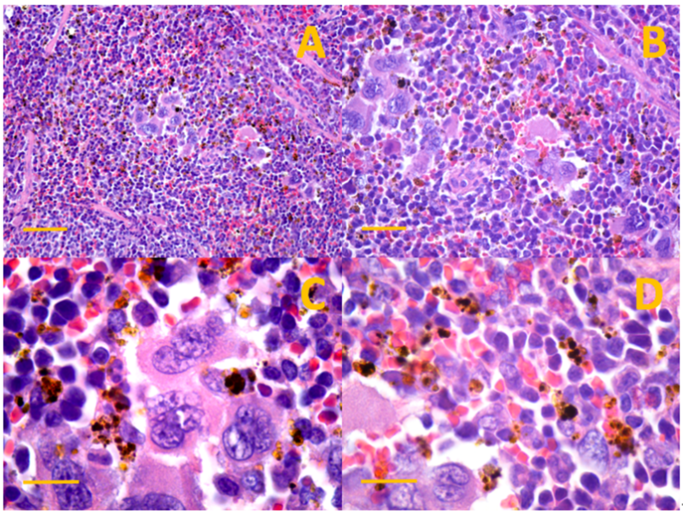

Figure S9. Transversal section through spleen collected at 14 days from mice injected with NP-DOX; hematoxilin-eosin staining, magnification: (a) 200× (measure bar of 50 µm); (b) 400× (measure bar of 25 µm); (c,d) 1000x (measure bar of 25 µm);

2.4.Hemocompatibility of NP-DOX

Hemocompatibility test was performed for different concentrations of NP-DOX nanoparticles in PBS, in order to evaluate the toxicity of the NPs on red blood cells. Results showed values of 0.525% hemolysis for the highest concentration employed in the study, which qualifies as a non-hemolytic effect.

3. References

1. Popescu, R.C.; Andronescu, E.; Vasile, B.Ş.; Truşcă, R.; Boldeiu, A.; Mogoantă, L.; Mogoșanu, G.D.; Temelie, M.; Radu, M.; Grumezescu, A.M.; Savu, D.I. Fabrication and Cytotoxicity of Gemcitabine- Fucntionalized Magnetite Nanoparticles. Molecules. **2017**, 22(7), 1080.
2. Temelie, M.; Popescu, R.C.; Cocioaba, D.; Vasile, B.S.; Savu, D. Biocompatibility study of magnetite nanoparticle synthesized using a green method. *RJP.*, **2018**, 63(7-8), 703.
3. Singh, N.P.; McCoy, M.T.; Tice, R.R.; Schneider, E.L. A simple technique for quantitation of low levels of DNA damage in individual cells. *Exp. Cell Biol.* **1988**, *175(1)*, 184-191.
